# Can we use biomarkers in combination with self-reports to strengthen the analysis of nutritional epidemiologic studies?

**DOI:** 10.1186/1742-5573-7-2

**Published:** 2010-01-20

**Authors:** Laurence S Freedman, Victor Kipnis, Arthur Schatzkin, Nataša Tasevska, Nancy Potischman

**Affiliations:** 1 Biostatistics Unit, Gertner Institute for Epidemiology, Tel Hashomer 52161, Israel; 2 Division of Cancer Prevention, National Cancer Institute, 6130 Executive Boulevard, EPN-3131, Bethesda, MD 20892-7354, USA; 3 Division of Cancer Epidemiology and Genetics, National Cancer Institute, 6120 Executive Boulevard, EPS-3040, Bethesda, MD 20892-7232, USA; 4 Division of Cancer Epidemiology and Genetics, National Cancer Institute, 6120 Executive Boulevard, EPS-3040, Bethesda, MD 20892-7232, USA; 5 Applied Research Program, Division of Cancer Control and Population Sciences, National Cancer Institute, 6130 Executive Boulevard, EPN-4008, Bethesda, MD 20892-7344, USA

## Abstract

Identifying diet-disease relationships in nutritional cohort studies is plagued by the measurement error in self-reported intakes.

The authors propose using biomarkers known to be correlated with dietary intake, so as to strengthen analyses of diet-disease hypotheses. The authors consider combining self-reported intakes and biomarker levels using principal components, Howe's method, or a joint statistical test of effects in a bivariate model. They compared the statistical power of these methods with that of conventional univariate analyses of self-reported intake or of biomarker level. They used computer simulation of different disease risk models, with input parameters based on data from the literature on the relationship between lutein intake and age-related macular degeneration.

The results showed that if the dietary effect on disease was fully mediated through the biomarker level, then the univariate analysis of the biomarker was the most powerful approach. However, combination methods, particularly principal components and Howe's method, were not greatly inferior in this situation, and were as good as, or better than, univariate biomarker analysis if mediation was only partial or non-existent. In some circumstances sample size requirements were reduced to 20-50% of those required for conventional analyses of self-reported intake.

The authors conclude that (i) including biomarker data in addition to the usual dietary data in a cohort could greatly strengthen the investigation of diet-disease relationships, and (ii) when the extent of mediation through the biomarker is unknown, use of principal components or Howe's method appears a good strategy.

## Introduction

One of the most challenging problems in nutritional epidemiology is that of measurement error in dietary reporting [1]. It is now recognized that in univariate models these errors attenuate estimated relative risks (RRs) and seriously reduce statistical power to detect diet-disease relationships. In multivariate models, measurement errors can cause under-estimation or over-estimation of RRs in an unpredictable manner [2].

Efforts to tackle the problem of dietary measurement error have included the use of biological markers of nutritional intake. One of their main uses has been the validation of self-report instruments. In this regard, two classes of biomarker have been identified: recovery and concentration biomarkers [3]. Recovery biomarkers are those based on recovery of certain products directly related to intake and not subject to substantial inter-individual differences in metabolism. Only a few examples exist, including the doubly-labeled water technique [4] for measuring energy expenditure (and hence indirectly energy intake), and 24-hour urinary nitrogen [5] for measuring protein intake. These biomarkers provide nearly unbiased measurements of intake, and are therefore extremely useful for validating self-report instruments.

Concentration biomarkers, such as serum carotenoids, are those that are related to dietary intake but not in as direct a manner as recovery biomarkers because their levels are the result of complex metabolic processes. In addition to dietary differences, there may be inter-individual differences in absorption, utilization, storage and excretion depending on host factors as well as environmental factors (e.g., oxidative stress) [6]. Yet, these biomarkers are useful as an integrated measure of nutritional status that can be related to disease. The use of these biomarkers for validating self-report instruments has been pervasive [7] but somewhat problematic since the biomarkers themselves do not represent a direct measure of intake. The most that can be gathered from such studies is the level of correlation between the self-report and the biomarker, but it is unclear how to use that correlation further.

Efforts to combine a "reference" self-report instrument with one or more concentration biomarkers to validate another self-report instrument [8-10] have relied on assumptions regarding the correlations between errors. See Rosner et al [11] for a recent review.

A second use of dietary biomarkers has been as stand-alone risk factors for disease, for example serum cholesterol for heart disease [12]. In this case, the finding of a strong relationship to disease led to efforts to modify the biomarker and thereby prevent the disease, either by dietary means [13] or by medication [14].

In this paper we examine a different use of dietary biomarkers, especially concentration biomarkers, namely, to strengthen tests of hypotheses regarding relationships between dietary intake and disease. The main setting for application is in prospective cohort studies, since biological samples can be taken before development of disease, minimizing the risks of reverse causation. In fact, in many nutritional epidemiology studies currently conducted, biological specimens, such as blood samples, are collected from the participants, often in the hope that they can be used to test as yet unidentified hypotheses.

We describe methods that can be used for combining self-reported dietary intake with a biomarker measurement, and use computer simulations with realistic inputs to demonstrate the levels of gain in efficiency that can be achieved from the combination. The simulations are based on the hypothesized diet-disease relationships between lutein and macular degeneration. (A second example, beta-cryptoxanthin and stomach cancer, is provided in Additional File 1: Appendix, Part B.) Our main aims are to bring this analytic strategy to the attention of epidemiologists, to quantify the gains in statistical power to detect the diet-disease relationship that could accrue from its use, and to discuss its limitations.

## Analysis

### The model

**Figure 1:**
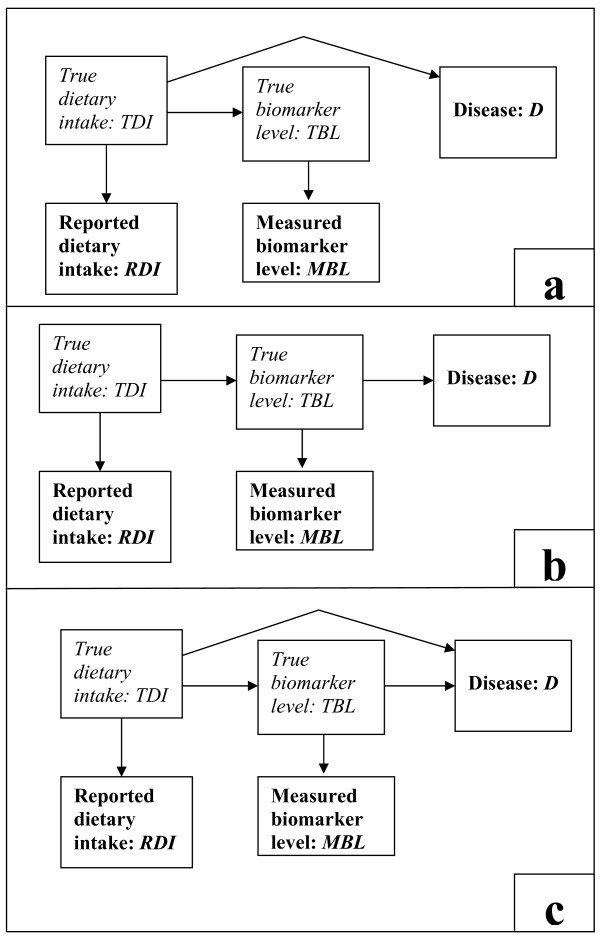
**A-C: Pathway diagrams for three versions of the model**. The variables typed in bold font are the observed variables; those in italic font are unobserved. Figure 1a represents the model where the diet effect on disease is not mediated by the biomarker (no mediation). Figure 1b represents the model where the diet effect is entirely mediated by the biomarker (full mediation). Figure 1c represents the general form of the model in which diet affects disease both through a pathway mediated by the biomarker and another pathway not mediated by the biomarker (partial mediation).

To elucidate some basic concepts involved in combining dietary reports and biomarkers, we propose a simple model in the form of a causal pathway diagram (Figure 1c). In the figure, the arrow from dietary intake to biomarker represents our assumption that true dietary intake causally affects the true biomarker level. Consequently, the two are correlated. Inasmuch as the reported intake and the measured biomarker level are correlated with their true values, they will also be correlated with each other.

The model represented by Figure 1c postulates that dietary intake affects disease through the biomarker and also through other pathways. This is the most general form of our model. For example, consider the potential effect of N-nitroso compounds (NOC) from red meat on colon cancer, with NOC-specific DNA adducts in exfoliated colonocytes as biomarkers of NOC exposure [15,16]. NOCs that reach the large intestine have a direct mutagenic effect on the colonic mucosa, resulting in formation of NOC-specific DNA adducts in the colonocytes, whereas absorbed NOCs can have a systematic effect on colonic tissue, acting as tissue specific carcinogens, directly or after metabolic activation [16].

We also consider two sub-models. In the first submodel, the biomarker of intake is not a determinant of disease; thus, in Figure 1a the arrow between the marker and disease is absent. For example, levels of urinary 3-methyl-histidine, a marker for red meat intake [17] are not thought to affect the risk of colon cancer, and would add nothing to the risk model if the true dietary intake were known. In the second submodel, dietary intake does not affect risk except through the biomarker (Figure 1b), and would add nothing to the risk model if the true biomarker level were known. An example is the dietary carotenoid intake, which is thought to affect skin melanoma entirely through the level of carotenoid in skin tissue [18].

When combining dietary reports with biomarkers in searching for nutrition-disease relationships we are using the biomarker in two different ways. Firstly, with regard to the diet-disease pathway not mediated through the biomarker, the biomarker acts as a correlate of dietary intake and helps to improve precision of our measure of dietary intake. Secondly, with regard to the diet-disease pathway through the biomarker, introduction of the biomarker naturally strengthens our ability to detect dietary effects through this pathway.

Finally, we note that Figure 1 does not include the possibility of confounding variables that causally affect the true biomarker level and independently the disease. As noted earlier, individual differences in metabolism and external factors can influence biomarker levels, so the presence of such confounders is a real possibility. We will proceed assuming that such confounding can be controlled for in the analysis, and elaborate on this important problem in the Discussion.

### The statistical model

Parallel to the model depicted in Figure 1, we define a statistical model. This will clarify the assumptions that are being made, and will also form the basis for generating simulated data and thereby studying the gains that can accrue from the combination methods that we will describe.

The model, depicted in Figure 1, can be represented mathematically by four inter-related statistical regression models:

(i) Biomarker-Diet: relating true biomarker level (*TBL*) to true dietary intake (*TDI*);

where the last term is distributed normally with mean zero and constant variance, independently of dietary intake. This part of the model describes the arrow from true dietary intake to true biomarker level in Figures 1a-c.

(ii) Biomarker Measurement: relating measured biomarker level (*MBL*) to true biomarker level;

where the last term is distributed normally with mean zero and constant variance, independently of true biomarker level. This is called the classical measurement error model [19], and implies that the measured level is an unbiased measure of the true level. This part of the model describes the arrow from true biomarker level to measured biomarker level in Figures 1a-c.

(iii) Dietary Intake Measurement: relating reported dietary intake (*RDI*) to true intake;

where the last term is distributed normally with mean zero and constant variance, independently of true intake. This part of the model describes the arrow from true dietary intake to reported dietary intake in Figures 1a-c.

(iv) Disease-Diet: relating disease (*D*) to true dietary intake and true biomarker level.

In this model, the coefficient *α*_2 _represents the effect of the biomarker level on disease, and describes the arrow from true biomarker level to disease in Figure 1c; the coefficient *α*_1 _represents the effect of diet on disease through pathways independent of the biomarker and describes the arrow from dietary intake to disease in Figure 1c. Assuming dietary intake causally affects biomarker level, the total effect of diet is the sum of *α*_1 _plus a multiple of *α*_2_. Setting *α*_2 _equal to zero is equivalent to deleting the arrow from biomarker to disease, as in Figure 1a. Setting *α*_1 _equal to zero is equivalent to deleting the arrow from dietary intake to disease, as in Figure 1b.

The main statistical assumptions underlying this four-part model and implied by Figure 1c are as follows.

1. Measurement errors in dietary intake are independent of disease, that is, non-differential.

2. Measurement errors in biomarker level are non-differential.

3. Measurement errors in dietary intake and in biomarker level are independent of each other. This seems reasonable since reporting errors are mostly cognitive whereas biomarker errors are mostly related to physiology or to laboratory conditions.

4. Any confounders of the biomarker-disease relationship and of the dietary intake-disease relationship have been controlled for (and are thus omitted from Figure 1). This is the strongest assumption, and we elaborate on it in the Discussion.

The assumptions regarding linearity of the regression models are not central to the main argument in this paper. If any of the regressions is non-linear then the dietary intake or biomarker level may be replaced by an appropriately transformed variable that will conform more closely to a linear relationship. Such transformation would not substantially change the results regarding statistical efficiency reported here.

### Statistical Methods of Relating Self-reported Intake and Biomarker Level to Disease

We assumed that: each cohort participant provides a self-reported dietary intake, a related biomarker measurement, and a binary disease outcome; self-report and biomarker values are transformed, if necessary, so their distributions are approximately normal; and relationships between dietary intake and disease are to be investigated using logistic regression. We considered 5 analytic approaches; the last three represent different ways of combining self-report and biomarker.

1. Univariate analysis (i.e. logistic regression with one explanatory variable) of self-reported intake;

2. Univariate analysis of biomarker level;

3. Bivariate analysis (i.e. logistic regression with two explanatory variables) of self-reported intake and biomarker level, testing the joint null hypothesis that the coefficients for self-reported intake and biomarker level are simultaneously zero. This joint hypothesis uniquely represents no association between diet and disease, assuming that dietary intake and biomarker do not affect disease in opposing directions.

4. Howe's method [20]. The two variables are grouped into k quantile groups, and the score j1 + j2 is calculated, where j1 is a participant's quantile for self-reported intake and j2 the quantile for biomarker level. The score is then used as the explanatory variable in the logistic regression. For k = 5, the range of possible scores is from 2 to 10. We studied the versions of the method with k = 3, 4, 5 and n (the sample size). With the last version, the score is the sum of the ranks of the two variables. We present results for this last version, as it was consistently the most efficient in our simulations.

5. Univariate analysis of the first principal components score. Principal components analysis [21] is performed on self-reported diet and biomarker level, the first principal component is formed and the scores of the first component are computed for each participant. Logistic regression is then conducted with the score as the explanatory variable. Principal components analysis is conducted on the correlation matrix, and the first principal component is the sum of the reported dietary intake and biomarker level weighted by the inverse of their respective standard deviations.

### Computer simulations

For simulating data, values of the coefficients in each of the four models described above must be specified, as well as the means and variances of the variables. Our aim was to quantify the potential gains in statistical power from using combined diet-biomarker analyses in realistic situations. We therefore chose two diet-disease hypotheses (to be described), and used results from the literature to determine the parameters for the simulation. The first hypothesis concerned dietary lutein and age-related macular degeneration (ARMD).

There is now considerable evidence that dietary lutein intake could reduce the incidence of ARMD [22]. Lutein, found in dark green, leafy vegetables is found in the macula and is thought to be protective though its antioxidant functions and as a blue light filter that protects underlying tissue from light damage [22,23]. Macular degeneration is an irreversible process that is a major cause of blindness in the elderly, and may be preventable through increased intake of lutein as well as zeaxanthin, by increasing the macular pigment [22].

The biomarker that we considered for dietary lutein intake was serum lutein. We considered two possible methods for self-report of lutein intake: a food frequency questionnaire (FFQ) or 6 repeated 24 hour recalls (24 HR). The FFQ is the instrument most commonly used to assess dietary intake in large prospective studies. Multiple 24 HR's are hypothesized to be more accurate than a FFQ [24] and are becoming more feasible to apply in large studies with the development of computerized versions [25].

To choose the parameters for the simulations, we scanned the literature for carotenoid feeding studies [26-29], cross-sectional studies of self-reported carotenoid intake and serum carotenoid levels [30-32], and epidemiologic studies relating carotenoid intake or serum levels to ARMD [33]. We also used unpublished data from the OPEN study [34]. The values of the parameters are shown in Table 1 and their determination is described in Additional File 1: Appendix, Part A.

**Table 1:** Parameters for the Lutein - Age Related Macular Degeneration Model

Model	Parameter	Value*				
Biomarker-Diet^a^	Intercept *β*_0_			5.29		
	Slope *β*_1_			0.60		
	Residual variance(*ε*_BL_)			0.10		
Biomarker Measurement^b^	Mean(*TBL*)			5.60		
	Variance(*TBL*)			0.19		
	Residual variance(*ε*_MBL_)			0.05		
			FFQ		6 × 24 HR	
	Intercept *γ*_0_		0.35		0.08	
	Slope *γ*_1_		0.71		0.84	
Dietary Intake Measurement^c^	Mean(*TDI*)			0.51		
	Variance(*TDI*)			0.25		
	Residual variance(*ε*_RDI_)		0.36		0.20	
Disease-Diet^d^		Model a		Model b		Model c
	Intercept *α*_0_	0.51		6.72		3.77
	Coefficient *α*_1_	-1.00		0.00		-0.48
	Coefficient *α*_2_	0.00		-1.20		-0.63

* Biomarker level is log transformed nmol/L; Dietary intake is log transformed mg/d. Parameter values are derived from data in references: Van het Hoff et al[26], Brevik et al[28], Delcourt et al[33], Dixon et al[30], Mares et al[31] and on unpublished data from the OPEN study[34], as described in Additional File 1: Appendix, Part A.

^a ^Parameters for regression of *TBL *on *TDI*

^b ^Parameters for regression of *MBL *on *TBL*

^c ^Parameters for regression of *RDI *on *TDI*

^d ^Parameters for regression of disease *D *on *TBL *and *TDI*

The second example, beta-cryptoxanthin and stomach cancer, is fully described in Additional File 1: Appendix, Part B.

We simulated cohort studies with 400 individuals, approximately half developing the disease, the other half remaining disease-free. One may regard these as representing nested case-control studies arising from cohort studies with a low incidence rate. To the data from each study, we applied the five statistical analyses listed previously.

After applying each analysis, we examined (a) whether a statistically significant relationship between disease and exposure was found at the 5% level on a two-sided test, and (b) the estimated RR between the 90^th ^and 10^th ^percentiles of the exposure variable distribution. For RR, Howe's method could not be compared with the other methods.

We examined 6 scenarios, three where the dietary report instrument was a FFQ and three where it was 6 repeats of a 24 HR. Each set of three scenarios comprised a disease risk model where the dietary effect on disease was not mediated through the biomarker (no mediation, as in Figure 1a), a model of full mediation (as in Figure 1b), and a model of partial mediation (as in Figure 1c). For each scenario we simulated 1000 cohort studies.

From the results on each scenario, we estimated statistical power as the proportion of statistically significant results, and the geometric mean of the RRs. We converted differences in statistical power to the ratio of sample size required to that required if a univariate analysis of reported dietary intake were used. This conversion was based on assuming that the test statistics were normally distributed.

### Results: Correlations between the exposure variables

The chosen model parameters shown in Table 1 gave rise to correlations between the exposure variables (Table 2). True dietary intake (*TDI*) was most strongly correlated with 6 × 24 HR reported intake (0.68), somewhat less strongly correlated (0.61) with observed serum lutein level (*MBL*), and least strongly correlated with FFQ reported dietary intake (0.51). True serum lutein level (*TBL*) was most strongly correlated with observed serum lutein level (0.89), and not very highly correlated with reported dietary intake (0.47 for 6 × 24 HR and 0.35 for FFQ).

**Table 2:** Lutein: Correlations Between Measurements Derived From the Chosen Model

		True diet lutein (*TDI*)	Reported diet lutein (*RDI*)		True serum lutein (*TBL*)	Measured serum lutein (*MBL*)
			FFQ	6 × 24 HR		
*TDI*		1.00				
*RDI*	FFQ	0.51	1.00			
	6 × 24 HR	0.68				
*TBL*		0.69	0.35	0.47	1.00	
*MBL*		0.61	0.31	0.42	0.89	1.00

### Simulation results

Results for scenarios where a FFQ was the dietary instrument are shown in Table 3. Estimated RRs (between the 90^th ^and 10^th ^percentiles of the measured exposure) were less than one, indicating the protective effect of lutein. For univariate analyses they varied between 0.32 and 0.64 according to the disease risk model and method of analysis. In most cases, the lower the RR in univariate analyses, the higher was the statistical power.

**Table 3:** Lutein and Age Related Macular Degeneration (ARMD), With Dietary Intake Assessed by FFQ: Standardized Relative Risks (RR*), Statistical Power and Relative Sample Size** (rss) Required for Various Analysis Strategies Under Different Disease Risk Models

Analysis Strategy		Disease Risk Model		
		(a) Not mediated through marker	(b) Mediated entirely through marker	(c) Partially mediated through marker
*RDI*^a^ (univariate)	RR Power (rss)	0.54 0.655 (1.00)	0.64 0.413 (1.00)	0.58 0.533 (1.00)
*MBL*^b^ (univariate)	RR Power (rss)	0.47 0.814 (0.68)	**0.32** **0.993 (0.16)**	0.38 0.952 (0.32)
Bivariate^c^	RR *RDI* RR *MBL* Power (rss)	0.65 0.54 0.839 (0.64)	0.90 0.33 0.986 (0.18)	0.76 0.41 0.941 (0.34)
Howe^d ^(ranks)	RR Power (rss)	0.42 0.891 (0.55)	0.36 0.958 (0.22)	0.38 0.948 (0.32)
Principal Components^e^	RR Power (rss)	**0.43** **0.904 (0.52)**	0.37 0.966 (0.21)	**0.39** **0.956 (0.31)**

* Standardized relative risk is defined as the RR between the 90^th ^and 10^th ^percentiles of the distribution.

**Bold type **indicates the most powerful method among the available choices investigated.

** Sample size requirement relative to the univariate RDI analysis.

^a ^Regression of ARMD on *RDI*

^b ^Regression of ARMD on *MBL*

^c ^Regression of ARMD on *RDI and MBL*

^d ^Regression of ARMD on combination of *RDI *and *MBL *using Howe's method

^e ^Regression of ARMD on combination of *RDI *and *MBL *using Principal Components

The univariate analysis of FFQ reported intake was less powerful than that of serum level. This was due to FFQ reported intake having a lower correlation with true dietary intake (r = 0.51) and with true serum level (0.35) than did measured serum level (0.61 and 0.89 respectively) (Table 2).

The combination methods generally performed much better than the univariate analysis of FFQ. Whether or not they improved on the univariate analysis of serum level depended on the disease risk model. When there was no mediation through the serum level, combination methods, especially principal components, produced moderate gains over the analysis of serum level alone. When there was partial mediation, principal components was only slightly more efficient than using serum level alone. When there was full mediation, then the univariate serum level analysis was optimal, although the principal components method was not much inferior.

Among the combination methods, principal components and Howe's method performed equally well. Bivariate analysis was less powerful than univariate analysis of serum level in the models with full and partial mediation, and less powerful than principal components and Howe's method in the models with no or partial mediation through the biomarker.

Projected sample size savings compared to univariate analysis of FFQ were substantial. Under full mediation the univariate serum analysis would require only 16% of the sample size needed for a dietary intake analysis, and under partial mediation 32%. Combination methods also gave substantial sample size savings, with the principal components yielding sample sizes between 21% (full mediation) and 52% (no mediation) of that required for univariate analysis of FFQ. In parallel with these sample size savings, observed RRs between the 10^th ^and 90^th ^percentiles were well below 0.5 using univariate serum level analysis or principal components, but above 0.5 for univariate analysis of FFQ.

Results where 6 24 HR's were the dietary instrument are shown in Table 4. These results show that when the dietary instrument was improved (correlation with true intake = 0.68), the gains from including the serum biomarker were less dramatic but still potentially useful. In the no mediation model, univariate analysis of 24 HR's gave more statistical power than univariate analysis of serum level, but was less powerful than the principal components method. The latter yielded a sample size requirement 77% that of the univariate analysis of 24 HR's.

**Table 4:** Lutein and Age Related Macular Degeneration (ARMD), With Dietary Intake Assessed by 6 24 HR's: Standardized Relative Risks (RR*), Statistical Power and Relative Sample Size** (rss) Required for Various Analysis Strategies Under Different Disease Risk Models

Analysis Strategy		Disease Risk Model		
		(a) Not mediated through marker	(b) Mediated entirely through marker	(c) Partially mediated through marker
*RDI*^a^ (univariate)	RR Power (rss)	0.43 0.890 (1.00)	0.55 0.629 (1.00)	0.49 0.810 (1.00)
*MBL*^b^ (univariate)	RR Power (rss)	0.47 0.829 (1.20)	**0.32** **0.995 (0.25)**	0.38 0.972 (0.54)
Bivariate^c^	RR *RDI* RR *MBL* Power (rss)	0.53 0.61 0.904 (0.95)	0.87 0.33 0.986 (0.30)	0.67 0.44 0.968 (0.55)
Howe^d ^(ranks)	RR Power (rss)	**0.38** **0.959 (0.74)**	0.34 0.985 (0.31)	0.35 0.974 (0.53)
Principal Components^e^	RR Power (rss)	0.39 0.952 (0.77)	0.35 0.984 (0.31)	**0.37** **0.977 (0.51)**

* Standardized relative risk is defined as the RR between the 90^th ^and 10^th ^percentiles of the distribution.

**Bold type **indicates the most powerful method among the available choices investigated.

** Sample size requirement relative to the univariate RDI analysis.

^a ^Regression of ARMD on *RDI*

^b ^Regression of ARMD on *MBL*

^c ^Regression of ARMD on *RDI and MBL*

^d ^Regression of ARMD on combination of *RDI *and *MBL *using Howe's method

^e ^Regression of ARMD on combination of *RDI *and *MBL *using Principal Components

When there was partial or full mediation through the serum level, then the power gains from univariate analysis of serum level and from the combination methods were substantial, with reduction of sample size to 30%-50%, relative to analysis of 24 HR's. However, in these models the combination methods did not perform better than the univariate serum level analysis.

The results for *β*-cryptoxanthin and stomach cancer were quite similar to those shown in Tables 3 and 4, and are described in Additional File 1: Appendix, Part B.

### Comments on the application of the method

The principal component or Howe's score has no recognized units, the first being a sum of two standardized scores, the second a sum of two rank scores. Other nutritional measures, such as "prudent diet" scores or the Healthy Eating Index, share this property. We propose that the principal components score be used as a more efficient first means of establishing the existence of a nutrition-disease relationship. Analyses that explore in more depth the relationships between dietary intake, biomarker level and disease risk will be motivated by such a positive result.

Markers that will be potentially useful in combination with dietary reports are those demonstrated in controlled feeding studies to be quantifiably modified by changes in diet. A causal relationship between marker and disease then implies that dietary intake will also affect disease, making it acceptable to combine the two measures. The level of the correlation between biomarker and reported dietary intake need not be high. In fact, from simulations not reported here, it appears that biomarkers are likely to be most helpful when reported intake is a poor measure of true intake, and in this situation the reported intake will also have low correlation with the biomarker. What is important is that the biomarker has a correlation with *true *intake that is similar, or preferably higher, than the correlation between reported intake and true intake. Some notion of whether this is so may be available from controlled feeding studies.

Another helpful characteristic is that the biomarker is not known to be affected by risk factors for the disease. This is related to the assumptions implicit in Figure 1. If there were risk factors that affected the biomarker, then the biomarker-disease association would be at least partly indirect. Factors that affect the metabolism of the dietary constituent or interact to change the biomarker levels, such as hypo-absorption or oxidative stress, may also have independent effects on the disease, and could thereby confound the diet-biomarker-disease relationship. In the worst case, modifying the biomarker level through diet change would not affect disease. The problem here is the familiar one of confounding that has been a consideration in previous studies of biomarkers and disease. In the event that a strong risk factor for the disease is known to affect the marker, that risk factor should at least be included in the disease risk model so as to avoid ascribing its effect as nutritional. For example, there is now some evidence that beta-cryptoxanthin is negatively associated with smoking [35], and smoking is a known risk factor for stomach cancer [36]. Thus, one should include smoking in the model linking disease to a combined measure of dietary intake/serum level of beta-cryptoxanthin. Thus, a price to pay for using a combined dietary intake-biomarker measure is the extra care needed in considering confounding factors since these could enter both through confounding with self-reported intake or through confounding with the biomarker level, and the uncertainty over whether introducing the biomarker has actually introduced an unwelcome confounder alongside the extra information on dietary intake. Here again, the higher is the correlation between the biomarker level and true intake, the more likely is the success of our proposed analytic strategy. For example, our analysis of the literature indicates an encouragingly high correlation of 0.69 between serum lutein and true dietary lutein intake (Table 2).

The practicality of including biomarker measurements in all participants in a large cohort study needs considering. As mentioned in the introduction, collecting biological samples from participants is no longer uncommon and their uses are manifold. Thus, while sample collection can be extremely expensive, the proposed approach may be feasible for studies with an already established "biobank". Furthermore, the sample size savings shown in Tables 3-4 indicate that adding a biomarker could lead to a two- to five-fold decrease in required sample size, which may partly offset the extra cost of collecting the specimens. Note that the analytic cost of the bioassays need not be prohibitive if analyses are based on a nested case-control design.

## Conclusion

We have demonstrated through computer simulation that including biomarkers in nutrition-disease analyses of prospective studies can substantially increase the statistical power for detecting a relationship and thereby reduce sample size requirements. The simulation model is relatively simple, but contains the statistical essentials necessary to analyze the problem and provide insight. An advantage of the simulations performed is that the input parameters are based on data from the literature.

Comparison of the results in Table 3 (FFQ) with those in Table 4 (6 × 24 HR's) show that the biomarker contributes incrementally less when the dietary report is more accurate, but the results under full and partial mediation models in Table 4 show that there remains room for further substantial increases in power from including biomarker data.

We will often be ignorant of the extent to which dietary intake effects are mediated by the biomarker. Therefore we need methods that perform well under different disease risk models. In our limited simulations, the principal components method and Howe's method both seemed to do this. They were superior to univariate biomarker analysis under the no mediation model and were not substantially inferior to that analysis under full mediation. Thus when we are ignorant of the extent to which the biomarker mediates the dietary effect, a combination approach using either of these methods would appear to be a good strategy. Howe's method has in fact been used, with apparently useful results, in two reports exploring carotenoid intake and prostate cancer [37,38]. In circumstances where we know that full mediation through the biomarker occurs, we should use the univariate analysis of the biomarker level rather than a combination method, as long as the biomarker measurement has relatively little measurement error.

In summary, the added information and statistical power demonstrated in our simulations suggest that including biomarker data in addition to the usual dietary data in a cohort could greatly strengthen the investigation of diet-disease relationships, and that, when the extent of mediation through the biomarker is unknown, use of a combination method such as principal components or Howe's method appears a good strategy.

## Abbreviations

ARMD: Age related macular degeneration; BL: Biomarker level; D: disease; DI: Dietary Intake; FFQ: Food frequency questionnaire; MBL: Measured biomarker level; OPEN: Observing Protein and Energy; RDI: Reported dietary intake; RR: Relative risk; 24 HR: 24 hour recall.

## Competing interests

The authors declare that they have no competing interests.

## Authors' contributions

LSF proposed this project, performed the computer simulations and wrote the major parts of the paper. VK made a major contribution to the statistical concepts and the design of the simulations. AS critiqued the general model and made a major contribution to the section on the application of the method to epidemiologic studies. NT made a major contribution to the reasoning behind the use of general model. NP identified the examples from the literature, critiqued the calculation of the parameters from the reported studies and contributed to the section on applications to epidemiological studies and to the conclusions. All authors have read and approved the final manuscript.

## Supplementary Material

Additional file 1 **Detailed calculations of the parameters for the simulations and further results**. Contains (a) data from the papers in the literature on lutein intake, serum lutein and macular degeneration and how they are used to calculate the parameters in the proposed statistical model; (b) data from the papers in the literature on beta-cryptoxantin intake, serum beta-cryptoxanthin and stomach cancer and how they are used to calculate the parameters in the proposed statistical model; and (c) tables presenting results of simulation of the model for beta-cryptoxanthin and stomach cancer. Click here for file

## References

[B1] Freudenheim JL Marshall JR The problem of profound mismeasurement and the power of epidemiologic studies of diet and cancer Nutr Cancer 1988 11 243 250 10.1080/01635588809513994 3217262

[B2] Day NE Wong MY Bingham S Khaw KT Luben R Michels KB Welch A Wareham NJ Correlated measurement error: implications for nutritional epidemiology Int J Epidemiol 2004 33 1373 1381 10.1093/ije/dyh138 15333617

[B3] Kaaks R Ferrari P Ciampi A Plummer M Riboli E Uses and limitations of statistical accounting for random error correlations, in the validation of dietary questionnaire assessments Public Health Nutr 2002 5 969 976 10.1079/PHN2002380 12638598

[B4] Schoeller DA Measurement error of energy expenditure in free-living humans by using doubly labeled water J Nutr 1988 118 1278 1289 3142975 10.1093/jn/118.11.1278

[B5] Bingham SA Cummings JH Urine nitrogen as an independent validatory measure of dietary intake: a study of nitrogen balance in individuals consuming their normal diet Am J Clin Nutr 1985 42 1276 1289 4072961 10.1093/ajcn/42.6.1276

[B6] Potischman N Biologic and Methodologic Issues for Nutritional Biomarkers J Nutr 2003 133 875S 880S 12612173 10.1093/jn/133.3.875S

[B7] Willett W Lenart E Willett W Reproducibility and validity of food-frequency questionnaires Nutritional Epidemiology 1998 2 New York, NY: Oxford University Press, Publishers

[B8] Kaaks R Riboli E Esteve J Van Kappel A Van Staveren W Estimating the accuracy of dietary questionnaire assessments: validation in terms of structural equation models Statist Med 1994 13 127 142 10.1002/sim.4780130204 8122049

[B9] Spiegelman D Zhao B Kim J Correlated errors in biased surrogates: study designs and methods for measurement error correction Statist Med 2005 24 1657 1682 10.1002/sim.2055 15736283

[B10] Fraser GE Butler TL Shavlik DJ Correlation between estimated and true dietary intakes: using two instrumental variables Ann Epidemiol 2005 15 509 518 10.1016/j.annepidem.2004.12.012 16029843

[B11] Rosner B Michels KB Chen YH Day NE Measurement error correction for nutritional exposures with correlated measurement error: Use of the method of triads in a longitudinal setting Statist Med 2008 27 3466 3489 10.1002/sim.3238 PMC3038790 18416440

[B12] Kannel WB Garcia MJ McNamara PM Pearson G Serum lipid precursors of coronary heart disease Hum Pathol 1971 2 129 151 10.1016/S0046-8177(71)80023-0 5095238

[B13] Dayton S Pearce ML Hashimoto S A controlled clinical trial of a diet high in unsaturated fat for preventing complications of atherosclerosis Circulation 1969 Suppl 2 1 63

[B14] Muldoon MF Manuck SB Matthew KA Lowering cholesterol concentrations and mortality: A quantitative review of primary prevention trials Br Med J 1990 301 309 314 10.1136/bmj.301.6747.309 PMC1663605 2144195

[B15] Cross AJ Sinha R Meat-related mutagens/carcinogens in the etiology of colorectal cancer Environ Mol Mutagen 2004 44 44 55 10.1002/em.20030 15199546

[B16] Lewin MH Bailey N Bandaletova T Bowman R Cross AJ Pollock J Shuker DE Bingham SA Red meat enhances the colonic formation of the DNA adduct O6-carboxymethyl guanine: implications for colorectal cancer risk Cancer Res 2006 66 1859 1865 10.1158/0008-5472.CAN-05-2237 16452248

[B17] Jacobson EA Newmark HL McKeown-Eyssen GE Bruce WR Excretion of 3-methylhistidine in urine as an estimate of meat consumption Nutr Rep Int 1983 27 689 697

[B18] Darvin ME Patzelt A Knorr F Blume-Peytavi U Sterry W Lademann J One-year study on the variation of carotenoid antioxidant substances in living human skin: influence of dietary supplementation and stress factors J Biomed Opt 2008 13 044028 10.1117/1.2952076 19021355

[B19] Carroll RJ Ruppert D Stefanski LA Crainiceanu CM Measurement Error in Nonlinear Models: A Modern Perspective 2006 2 Boca Raton, FL: Chapman and Hall/CRC Press

[B20] Howe GR The use of polytomous dual response data to increase power in case-control studies: an application to the association between dietary fat and breast cancer J Chron Dis 1985 38 663 670 10.1016/0021-9681(85)90020-7 4019703

[B21] Jolliffe IT Principal Components Analysis 2002 2 New York, NY: Springer, Publishers

[B22] Krinksy NI Landrum JT Bone RZ Biologic mechanisms of the protective role of lutein and zeaxanthin in the eye Ann Rev Nutr 2003 23 171 201 10.1146/annurev.nutr.23.011702.073307 12626691

[B23] Renzi LM Johnson EJ Lutein and age-related ocular disorders in the older adult: a review J Nutr Elder 2007 26 130 157 10.1300/j052v26n03_07 18285296

[B24] Kipnis V Subar AF Midthune D Freedman LS Ballard-Barbash R Troiano RP Bingham S Schoeller DA Schatzkin A Carroll RJ The structure of dietary measurement error: results of the OPEN biomarker study Am J Epidemiol 2003 158 14 21 10.1093/aje/kwg091 12835281

[B25] Subar AF Thompson FE Potischman N Forsyth BH Buday R Richards D McNutt S Hull SG Guenther PM Schatzkin A Baranowski T Formative research of a quick list for an Automated Self-Administered 24-hour dietary recall J Am Diet Assoc 2007 107 1002 1007 10.1016/j.jada.2007.03.007 17524721

[B26] Van het Hoff KH Brouwer IA West CE Haddeman E Steegers-Theunissen RP van Dusseldorp M Weststrate JA Eskes TK Hautvast JG Bioavailability of lutein from vegetables is five times higher than that of beta-carotene Am J Clin Nutr 1999 70 261 268 10426704 10.1093/ajcn.70.2.261

[B27] Muller H Bub A Watzl B Rechkemmer G Plasma concentrations of carotenoids in healthy volunteers after intervention with carotenoid-rich foods Europ J Nutr 1999 38 35 44 10.1007/s003940050044 10338686

[B28] Brevik A Andersen LF Karlsen A Trygg KU Blomhoff R Drevon CA Six carotenoids in plasma used to assess recommended intake of fruits and vegetables in a controlled feeding study Eur J Clin Nutr 2004 58 1166 1173 10.1038/sj.ejcn.1601945 15054430

[B29] Bowen PE Garg V Stacewicz-Sapuntzakis M Yelton L Schreiner RS Variability of serum carotenoids in response to controlled diets containing six servings of fruits and vegetables per day Ann NY Acad Sci 1993 691 241 243 10.1111/j.1749-6632.1993.tb26182.x 8129301

[B30] Dixon LB Subar AF Wideroff W Thompson FE Kalhle LL Potischman N Carotenoid and tocopherol estimates from the NCI Diet History Questionnaire are valid compared with multiple recalls and serum biomarkers J Nutr 2006 136 3054 3061 17116719 10.1093/jn/136.12.3054

[B31] Mares JA LaRowe TL Snodderly DM Moeller SM Gruber MJ Klein ML Wooten BR Johnson EJ Chappell RJ CAREDS Macular Pigment Study Group and Investigators Predictors of optical density of lutein and zeaxanthin in retinas of older women in the Carotenoids in Age-Related Eye Disease Study, an ancillary study of the Women's Health Initiative Am J Clin Nutr 2006 84 1107 1122 17093164 10.1093/ajcn/84.5.1107

[B32] Gruber M Chappell R Millen A LaRowe T Moeller SM Iannaccone A Kritchevsky SB Mares J Correlates of serum lutein + zeaxanthin: findings from the third National Health and Nutrition Examination Survey J Nutr 2004 134 2387 2394 15333733 10.1093/jn/134.9.2387

[B33] Delcourt C Carriere I Delage M Barberger-Gateau P Schalch W the POLA Study Group Plasma lutein and zeaxanthin and other carotenoids as modifiable risk factors for age-related maculopathy and cataract: the POLA Study Invest Opthalmol Vis Sci 2006 47 2329 2335 10.1167/iovs.05-1235 16723441

[B34] Subar AF Kipnis V Troiano RP Midthune D Schoeller DA Bingham S Sharbaugh CO Trabulsi J Runswick S Ballard-Barbash R Sunshine J Schatzkin A Using intake biomarkers to evaluate the extent of dietary misreporting in a large sample of adults: the OPEN study Am J Epidemiol 2003 158 1 13 10.1093/aje/kwg092 12835280

[B35] Stram DO Yuan JM Chan KK Gao YT Ross RK Yu MC Beta-cryptoxanthin and lung cancerin Shanghai, China, - an examination of potential confounding with cigarette smoking using urinary cotinine as a biomarker for true tobacco exposure Nutr Cancer 2007 57 123 129 17571944 10.1080/01635580701273998

[B36] US Surgeon General and Centers for Disease Control and Prevention The health consequences of smoking: a report of the Surgeon General. [Atlanta, Ga.] 2004 Dept. of Health and Human Services, Centers for Disease Control and Prevention, National Center for Chronic Disease Prevention and Health Promotion, Office on Smoking and Health; Washington, D.C

[B37] Wu K Erdman JW Schwartz SJ Platz EA Leitzmann M Clinton SK DeGroff V Willett WC Giovannucci E Plasma and dietary carotenoids, and the risk of prostate cancer: a nested case-control study Cancer Epidemiol Biomarker Prev 2004 13 260 269 10.1158/1055-9965.EPI-03-0012 14973107

[B38] Mikhak B Hunter DJ Spiegelman D Platz EA Wu K Erdman JW Jr Giovannucci E Manganese superoxide dismutase (MnSOD) gene polymorphism, interactions with carotenoid levels, and prostate cancer risk Carcinogenesis 2008 29 2335 2340 10.1093/carcin/bgn212 18784358 PMC2722865

