# Supplementary material for: Can we use biomarkers in combination with self-reports to strengthen the analysis of nutritional epidemiologic studies?
Source: Epidemiol Perspect Innov. 2010 Jan 20;7:2. doi: 10.1186/1742-5573-7-2 (PMC2841599; doi:10.1186/1742-5573-7-2)
Supplement: Additional file 1 — Detailed calculations of the parameters for the simulations and further results. Contains (a) data from the papers in the literature on lutein intake, serum lutein and macular degeneration and how they are used to calculate the parameters in the proposed statistical model; (b) data from the papers in the literature on beta-cryptoxantin intake, serum beta-cryptoxanthin and stomach cancer and how they are used to calculate the parameters in the proposed statistical model; and (c) tables presenting results of simulation of the model for beta-cryptoxanthin and stomach cancer. [file 1742-5573-7-2-S1.DOC]

Can we use biomarkers in combination with self-reports to strengthen the analysis of nutritional epidemiologic studies? –

Appendix: Detailed calculations of the parameters for the simulations and further results

**General:**

For the purposes of estimating variances, slopes and correlations, data from the literature were drawn from measurements on both genders, on the assumption that after a log transformation these parameters do not differ substantially according to gender. For the purposes of estimating means and intercepts, data were taken only from measurements on women.

**A: Lutein – Age Related Macular Degeneration Model**

Lutein co-elutes with zeaxanthin in many laboratory assays so some of the reports used in these analyses use the values for lutein/zeaxanthin as the lutein value. In OPEN, the concentration of zeaxanthin was approximately 0.25 that of lutein and the correlation was 0.71.

**A1. The Biomarker-Diet Model**:

$BL=\beta_{0}+\beta_{1}DI+\varepsilon_{BL}$ ,

where *BL* is log true serum lutein (nmol/L) and *DI* is log true lutein intake (mg/d).

(a) Parameter $\beta_{1}$ was taken from the feeding study of Van het Hoff et al^26^. They reported mean serum levels of 260 and 610 nmol/L among persons fed an average of 2.7 and 10.7 mg/d of lutein respectively. The slope between the lower dose and the higher dose on the log scale is 0.62. We took the value 0.6 for the slope. So $\beta_{1}$= 0.60.

(b) Parameter var($\varepsilon_{BL}$) was taken from the feeding study reported by Brevik et al^28^ who reported mean lutein levels and standard deviations (SD) for 40 individuals on a high vegetable and low vegetable diet in a crossover design. Assuming a lognormal distribution and little or no variation in lutein intake among the subjects in this group, var($\varepsilon_{BL}$) = ln(1+*CV*^2^), where *CV* is the coefficient of variation = SD/mean. An average CV of 0.33 was obtained in the first period when compliance was presumably highest. Thus an estimate var($\varepsilon_{BL}$) = 0.10 was obtained. The study by Van vet Hoff et al^26^ did not report the variance of the total lutein serum level but only that of baseline and change, so that we could not estimate var($\varepsilon_{BL}$) from this study.

(c) In the serum lutein measurement error model below, we assume the mean of *BL* to be 5.60 (See Section A2). Furthermore, in the dietary lutein measurement error model below (See Section A3.1), we assume mean *DI* to be 0.51.

Therefore the value for$\beta_{0}$is given by 5.60 – 0.6 x 0.51 = 5.29.

**A2. The Biomarker Measurement Model**:

$$MBL=BL+\varepsilon_{MBL}$$

where *MBL* is the logarithm of measured serum lutein level.

(a) Mean(*BL*): The median measured serum level of lutein (Table 2 of reference^33^) was 0.27 μmol/L = 270 nmol/L. Therefore log measured serum level (*MBL*) has a mean of ln(270) = 5.60, assuming that measured serum level has a lognormal distribution. According to the assumption that *MBL* is an unbiased measurement of *BL*, it follows that *BL* also has a mean of 5.60.

(b) Variance(*BL*):

(i) The variance of *MBL* can be obtained directly from the percentiles of measured serum lutein in Delcourt et al^33^ (Table 2 of that reference), leading to a variance of 0.24. Similar values are obtained from the OPEN study.

(ii) The within-person variance, var($\varepsilon_{MBL}$), for *MBL* is calculated from data of Dixon et al^30^. They reported a within-person correlation on repeated serum measurements of 0.84 for 86 women and 0.78 for 44 men. We took the correlation to be 0.80. This correlation is equal to the variance of *BL* divided by the variance of *MBL*, implying that var(*BL*) = var(*MBL*) x 0.80 = 0.24 x 0.80 = 0.19.

(iii) As a corollary, var($\varepsilon_{MBL}$) = var(*MBL*) – var(*BL*) = 0.24 – 0.19 = 0.05.

**A3. The Dietary Intake Measurement Model**:

$$RDI=\gamma_{0}+\gamma_{1}DI+\varepsilon_{RDI}$$

where *RDI* is the logarithm of self-reported lutein intake. We consider first self-report by a single food frequency questionnaire and secondly by 6 repeated 24 hour recalls. Parameters are calculated from various sources.

**A3.1 Food frequency questionnaire**

(a) Using the fact that var(*BL*) = 0.19 (see the serum lutein measurement model above), and also the values of the parameters for the biomarker-intake model above:

var(*DI*) = (var(*BL*) – var($\varepsilon_{BL}$))/$\beta_{1}^{2}$= (0.19 - 0.10)/(0.36) = 0.25.

(b) Mean *RDI* is taken from Mares et al^31^. They give the median reported intake of lutein/zeaxanthin to be 2.04 mg/d (their Table 2). Assuming a lognormal distribution of intake, this implies that mean *RDI* is exp(2.04) = 0.71. A similar value is obtained from Table 3 of Dixon et al^30^ and from the OPEN study.

(c) var(*RDI*) is estimated from the same Table 2 of Mares et al^31^ to be (ln(90^th^ percentile /10^th^ percentile)/2.56)^2^ = (ln(4.40/0.73)/2.56)^2^ = 0.49, assuming a lognormal distribution. A similar value is obtained from Table 3 of Dixon et al^30^ and also from the OPEN study.

(d) The slope parameter $\gamma_{1}$ was estimated on the basis of the observed correlation between *MBL* and *RDI*. In the OPEN study this correlation was taken as 0.31 (0.30 in women and 0.32 in men). Under our model assumptions, this correlation is equal to $\frac{\beta_{1}\gamma_{1}\mathrm{var}(DI)}{\sqrt{\mathrm{var}(MBL)\mathrm{var}(RDI)}}$. Thus, $\gamma_{1}$was estimated from the equation:

0.31 = $\frac{\beta_{1}\gamma_{1}\mathrm{var}(DI)}{\sqrt{\mathrm{var}(MBL)\mathrm{var}(RDI)}}$, which led to $\gamma_{1}$=0.71.

(e) The intercept$\gamma_{0}$was obtained by assuming that a 24 hour recall is unbiased at the group level for *DI*, so that mean *DI* = 0.51 (see below in section A3.2). To calculate $\gamma_{0}$, the equation is:

Mean *RDI* = $\gamma_{0}$ + $\gamma_{1}$ x Mean *DI*, leading to 0.71 = $\gamma_{0}$+ 0.71 x 0.51.

So,$\gamma_{0}$= 0.35.

(f) The variance of the residual var($\varepsilon_{RDI}$) was then obtained by subtraction.

var($\varepsilon_{RDI}$) = var(*RDI*) -$\gamma_{1}^{2}$ x var(*DI*) = 0.49 – 0.71^2^ x 0.25 = 0.36

**A3.2 24-hour recalls**

(a) Mean *RDI* is based on unpublished data from the OPEN study. The geometric mean reported intake of lutein/zeaxanthin in 218 women was 1.67 mg/d. Using this value, the mean *RDI* is ln(1.67) = 0.51.

(b) The variance of *RDI* was based on the group of 218 women and 235 men participants in OPEN and was calculated as 0.61.

(c) The slope parameter $\gamma_{1}$ was estimated on the basis of the observed correlation between *MBL* and *RDI*. In the OPEN study this correlation was taken as 0.33 (0.33 in women and 0.34 in men). Under our model assumptions, this correlation is equal to $\frac{\beta_{1}\gamma_{1}\mathrm{var}(DI)}{\sqrt{\mathrm{var}(MBL)\mathrm{var}(RDI)}}$. Thus, $\gamma_{1}$was estimated from the equation:

0.33 = $\frac{\beta_{1}\gamma_{1}\mathrm{var}(DI)}{\sqrt{\mathrm{var}(MBL)\mathrm{var}(RDI)}}$, which led to $\gamma_{1}$=0.84.

(d) The variance of the residual var($\varepsilon_{RDI}$) was then obtained by subtraction.

var($\varepsilon_{RDI}$) = var(*RDI*) -$\gamma_{1}^{2}$ x var(*DI*) = 0.61 – 0.84^2^ x 0.25 = 0.43

Now the OPEN study had only 2 repeated 24 hour recalls. However, we are investigating the use of 6 repeated administrations of the instrument, so we needed to adjust the residual variance var($\varepsilon_{RDI}$) for this. We assumed that the residual variance on a 24 hour recall is comprised of 80% within-person variation and 20% subject specific error. This assumption came from data on energy and protein gathered in the OPEN study^39^. When we move from 2 x 24HR’s to 6 x 24HR’s we need to divide the within-person variance by 6/2 = 3. Accordingly, the residual variance with 6 x 24 hour recalls = 0.43 x 0.2 + 0.43 x 0.8/3 = 0.20. Hence for 6 x 24HR’s, var($\varepsilon_{RDI}$) = 0.20.

(f) The intercept$\gamma_{0}$was obtained as follows:

Mean *RDI* = $\gamma_{0}$ + $\gamma_{1}$ x Mean *DI*, leading to 0.51 = $\gamma_{0}$+ 0.84 x 0.51.

So,$\gamma_{0}$= 0.08.

**A4. Disease Model**:

$$\text{logit}(\Pr(D=1))=\alpha_{0}+\alpha_{1}DI+\alpha_{2}BL$$

where *D* is disease (0,1).

Data from a study by Delcourt et al^33^ indicate that $\alpha_{2}$ ~ -1.15 when *DI* is omitted from the model. We obtained this from their Table 2, which shows a RR of 0.31 between the lowest quintile and highest quintile of measured serum lutein. Estimating the median *MBL*’s in these two groups to be 4.98 and 6.23 respectively (using the assumption of a lognormal distribution) we get the slope of log RR on *MBL* to be -0.94. Adjusting for measurement error in the serum level (dividing the estimate by 0.19/0.24) we get a slope of −1.19 for *BL*. We rounded this to −1.2.

Scenario 1: We do not know how the individual components *DI* and *BL* relate simultaneously to ARMD. We first constructed a scenario in which all the effect on disease is mediated through the dietary intake. We chose a coefficient for *DI* which would lead to the observation that $\alpha_{2}$ ~ −1.2 if *DI* were omitted from the model. Approximately, $\alpha_{1}$ = $\alpha_{2}$ x var(*BL*) / ($\beta_{1}$x var(*DI*)) = −1.2 x 0.19/(0.60 x 0.37) = −1.03. We rounded this value to $\alpha_{1}$=−1.0.

Thus our first scenario had $\alpha_{1}$=-1.0 and $\alpha_{2}$=0. We chose $\alpha_{0}$ so that that approximately 50% of the participants were controls and 50% cases. In this scenario $\alpha_{0}$=0.51.

Scenario 2:

We constructed a second scenario in which all the effect on disease is mediated through the biological marker level. Here we applied the data from Delcourt et al^33^ directly and we took $\alpha_{0}$=6.72, $\alpha_{1}$=0 and $\alpha_{2}$=−1.2.

Scenario 3:

We constructed a third scenario where the effect of disease is mediated approximately equally through the dietary intake and biological marker. We took $\alpha_{0}$=3.77, $\alpha_{1}$=−0.48 and $\alpha_{2}$=−0.63, the two coefficients being approximately half their values in Scenarios 1 and 2.

**B: Beta-cryptoxanthin – Stomach Cancer Model**

**B0: Background**

Jenab et al^40^ report a strong negative association between serum β-cryptoxanthin levels and the incidence of gastric adenocarcinoma in a multinational nested case-control study within the European Prospective Investigation into Cancer and Nutrition (EPIC), conducted in 10 countries, which evaluated blood carotenoids and tocopherols in relation to incident gastric adenocarcinoma. The strongest associations were observed for β-cryptoxanthin (OR 0.53, 95% CI 0.30-0.94) and zeaxanthin (OR=0.39, 95% CI=0.22-0.69) among the carotenoids. These two carotenoids are xanthophyls that have stronger associations with intake than other carotenoids^30,34^ (unpublished data from the study described in the latter reference). We chose to study β-cryptoxanthin, since it is more difficult to find the necessary published data for serum zeaxanthin, which is combined with lutein in many reports as they co-elute in laboratory analyses.

The biomarker that we consider for dietary β-cryptoxanthin intake is serum β-cryptoxanthin. As with the lutein example, we consider two possible methods for self-report of β-cryptoxanthin intake: a food frequency questionnaire (FFQ) or 6 repeated 24 hour recalls (24HR).

**B1. The Biomarker-Diet Model**:

$$BL=\beta_{0}+\beta_{1}DI+\varepsilon_{BL}$$

where *BL* is log serum β-cryptoxanthin in nmol/L and *DI* is log intake of β-cryptoxanthin in mg/d.

(a) Parameter $\beta_{1}$ was taken from Van het Hoff et al^26^. After adjusting their results so that baseline values were equal in the comparison groups, we calculated mean serum levels of 250, 160 and 420 nmol/L among persons fed an average of <0.2, 0.21 and 0.84 mg/d of β-cryptoxanthin respectively. We combined the two lower dose groups to form one group with average intake of 0.14 mg/dl and adjusted mean serum level of 190 nmol/L. The slope between this lower dose group and the higher dose group on the log scale was then 0.44. We rounded the value to 0.45.

(b) Parameter var($\varepsilon_{BL}$) was taken from the feeding study reported by Brevik et al^28^ who reported mean β-cryptoxanthin levels and standard deviations (SD) for 40 individuals on a high vegetable and low vegetable diet in a crossover design. Assuming a lognormal distribution and little or no variation in β-cryptoxanthin intake among the subjects in this group, var($\varepsilon_{BL}$) = ln(1+*CV*^2^), where *CV* is the coefficient of variation = SD/mean. An average CV of 0.39 was obtained in the first period when compliance was presumably highest. Thus an estimate var($\varepsilon_{BL}$) = 0.14 was obtained. The study by Van vet Hoff et al^26^ did not report the variance of the β-cryptoxanthin serum level but only that of baseline and change, so that we could not estimate var($\varepsilon_{BL}$) from this study.

(c) In the serum β-cryptoxanthin measurement error model below, we assume the mean of *BL* to be 5.30. Furthermore, in the dietary β-cryptoxanthin measurement error model below, we assume mean *DI* to be -2.85.

Therefore the value for$\beta_{0}$is given by 5.30 – 0.45 x (-2.85) = 6.58.

**B2 The Biomarker Measurement Model**:

$$MBL=BL+\varepsilon_{BL}$$

where *MBL* is the logarithm of measured serum β-cryptoxanthin level (classical measurement error model).

(a) Mean log serum level: Geometric mean serum level of β-cryptoxanthin (OPEN study) was 11.0μg/dl for women, which translates to 199nmol/L. Therefore log measured serum level (*MBL*) has a mean of ln(199) = 5.30, assuming that measured serum level has a lognormal distribution. According to the assumption that *MBL* is an unbiased measurement of *BL*, it follows that *BL* also has a mean of 5.30.

(b) Variance log serum level:

(i) The *CV* of serum β-cryptoxanthin from the EATS study^30^ in 86 women and 44 men free-living individuals was 0.78 for women and 0.59 for men, leading to var(*MBL*) = 0.48 for women and 0.30 for men. For approximately 3000 individuals from a variety of European populations in the EPIC study^41^, the *CV*’s were 0.84 for men and 0.80 for women, leading to var(*MBL*) =0.54 for men and 0.49 for women. Results from the OPEN study on 475 individuals lead to estimates of var(*MBL*) = 0.32 for men and 0.40 for women. We took the value 0.45, which is higher than the values for OPEN and lower than the values for EPIC. Therefore, var(*MBL*) =0.45.

(ii) The within-person variance, var(*u*), for *MBL* is calculated from data of Dixon et al^30^. They reported a within-person correlation on repeated serum β-cryptoxanthin measurements of 0.82 for women and 0.84 for men. We assumed a correlation of 0.83. This correlation is approximately equal to the variance of *BL* divided by the variance of *MBL*. This implies that var(*BL*) = var(*MBL*) x 0.83 = 0.45 x 0.83 = 0.37.

(iii) As a corollary, var(*u*) = var(*MSC*) – var(*SC*) = 0.45 – 0.37 = 0.08.

**B3. The Dietary Intake Measurement Model**:

$$RDI=\gamma_{0}+\gamma_{1}DI+\varepsilon_{RDI}$$

where *RDI* is the logarithm of self-reported β-cryptoxanthin intake. We consider first self-report by a single food frequency questionnaire and secondly by 6 repeated 24 hour recalls. Parameters are calculated from various sources.

**B3.1 Food frequency questionnaire**

(a) Mean *RDI* reported on a food frequency questionnaire was based on data from Dixon et al^30^ and from the OPEN study. Dixon et al gave the geometric mean reported intake of β-cryptoxanthin to be 0.13 mg/d among the 86 women (their Table 3). The 215 women in the OPEN study had a geometric mean reported intake of total cryptoxanthin equal to 0.139 mg/dl. Assuming that β-cryptoxanthin makes up approximately 75% of total cryptoxanthin, the OPEN study result led to an estimate of 0.104 mg/dl for the geometric mean for β-crypto. From these studies we took the geometric mean to be 0.11 mg/dl (giving more weight to OPEN since it is a larger study). This led to the value of ln(0.11) = -2.21 for mean *RDI*.

(b) var(*RDI*) is estimated from Dixon et al’s^30^ Table 3 to be sample size x (ln(95^th^ percentile /5^th^ percentile)/3.92)^2^ = 86 x (ln(.154/.109)/3.92)^2^ = 0.67, assuming a lognormal distribution. An almost identical estimate (0.66) is obtained from the OPEN study. We assumed the value var(*RDI*)=0.67.

(c) Using the assumption that var(*BL*) = 0.37 (see Section B2), and the parameters of the biomarker-diet model above (Section B1):

var(*DI*) = (var(*BL*) – var($\varepsilon_{BL}$))/$\beta_{1}^{2}$= (0.37 - 0.14)/(0.45^2^) = 1.14.

(d) The slope parameter $\gamma_{1}$ was estimated on the basis of the observed correlation between *MBL* and *RDI*. In the OPEN study this correlation was taken as 0.50 (0.52 in women and 0.49 in men). Under our model assumptions, this correlation is equal to $\frac{\beta_{1}\gamma_{1}\mathrm{var}(DI)}{\sqrt{\mathrm{var}(MBL)\mathrm{var}(RDI)}}$. Thus, $\gamma_{1}$was estimated from the equation:

0.50 = $\frac{\beta_{1}\gamma_{1}\mathrm{var}(DI)}{\sqrt{\mathrm{var}(MBL)\mathrm{var}(RDI)}}$, which led to $\gamma_{1}$=0.54.

(e) The intercept$\gamma_{0}$was obtained by assuming that *DI* was reported without bias on the 24 hour recall assessments (see section B3.2 below). Using this assumption, mean *DI* = ln(0.060) = -2.81.

The equation for $\gamma_{0}$ is:

Mean *RDI* = $\gamma_{0}$ + $\gamma_{1}$ x Mean *DI*, leading to -2.11 = $\gamma_{0}$+ 0.54 x -2.81.

So,$\gamma_{0}$= -0.59.

(f) The variance of the residual var($\varepsilon_{RDI}$) was then obtained by subtraction.

var($\varepsilon_{RDI}$) = var(*RDI*) -$\gamma_{1}^{2}$ x var(*DI*) = 0.67 – 0.54^2^ x 1.14 = 0.34

**B3.2 24 hour recalls**

(a) Mean *RDI* reported on a 24 hour recall was based on data from Dixon et al^30^ and from the OPEN study. Dixon et al gave the geometric mean reported intake of β-cryptoxanthin to be 0.058 mg/d among the 86 women (their Table 3). The 215 women in the OPEN study had a geometric mean reported intake of total cryptoxanthin equal to 0.0832 mg/dl. Assuming that β-cryptoxanthin makes up approximately 75% of total cryptoxanthin, the OPEN study result led to an estimate of 0.062 mg/d for the geometric mean for β-crypto. From these studies we took the geometric mean to be 0.060 mg/dl (giving more weight to OPEN since it is a larger study). This led to the value of ln(0.060) = -2.81 for mean *RDI*.

(b) var(*RDI*) was estimated from the OPEN study to be 2.91 and 2.94 for women and men respectively, using an average of 2 24HR’s. We took as the value in our study, var(*RDI*) = 2.90

(c) The slope parameter $\gamma_{1}$ was estimated on the basis of the observed correlation between *MBL* and *RDI*. In the OPEN study this correlation was taken as 0.43 (0.47 in women and 0.39 in men). Under our model assumptions, this correlation is equal to $\frac{\beta_{1}\gamma_{1}\mathrm{var}(DI)}{\sqrt{\mathrm{var}(MBL)\mathrm{var}(RDI)}}$. Thus, $\gamma_{1}$was estimated from the equation:

0.43 = $\frac{\beta_{1}\gamma_{1}\mathrm{var}(DI)}{\sqrt{\mathrm{var}(MBL)\mathrm{var}(RDI)}}$, which led to $\gamma_{1}$=0.96.

(d) The variance of the residual var($\varepsilon_{RDI}$) was then obtained by subtraction.

var($\varepsilon_{RDI}$) = var(*RDI*) -$\gamma_{1}^{2}$ x var(*DI*) = 2.90 – 0.96^2^ x 1.14 = 1.85

Now the OPEN study had only 2 repeated 24 hour recalls. However, we are investigating the use of 6 repeated administrations of the instrument, so we needed to adjust the residual variance var($\varepsilon_{RDI}$) for this. We assumed that the residual variance on a 24 hour recall is comprised of 80% within-person variation and 20% subject specific error. This assumption came from data on energy and protein gathered in the OPEN study^39^. When we move from 2 x 24HR’s to 6 x 24HR’s we need to divide the within-person variance by 6/2 = 3. Accordingly, the residual variance with 6 x 24 hour recalls = 1.85 x 0.2 + 1.85 x 0.8/3 = 0.86. Hence for 6 x 24HR’s, var($\varepsilon_{RDI}$) = 0.86.

(e) Now the OPEN study had only 2 repeated 24 hour recalls. However, we are investigating the use of 6 repeated administrations of the instrument, so we need to divide the residual by 6/2=3. Hence for 6 x 24HR’s, var($\varepsilon_{RDI}$) = 0.38/3 = 0.13.

(f) The intercept$\gamma_{0}$was obtained as follows:

Mean *RDI* = $\gamma_{0}$ + $\gamma_{1}$ x Mean *DI*, leading to -2.81 = $\gamma_{0}$+ 0.96 x -2.81.

So,$\gamma_{0}$= -0.11.

**B4. Disease Model**:

$$\text{logit}(D)=\alpha_{0}+\alpha_{1}DI+\alpha_{2}BL$$

where *D* is stomach cancer (0,1); *DI* is the logarithm of true usual β-cryptoxanthin intake (mg/d); and *BL* is the logarithm of true serum β-cryptoxanthin level (nmol/L).

Parameter values:

Data from a study by Jenab et al^40^ indicated that $\alpha_{2}$ ~ -0.50 when *DC* is omitted from the model. This was calculated from their Table 3, which showed a RR of 0.53 between the lowest quartile and highest quartile of measured serum β-crypto. The RR had been adjusted for smoking, a potential confounder. Estimating the median *MBL*’s in these two groups to be 1.68 and 3.27 respectively (using the assumption of a lognormal distribution) we get the slope of log RR on *MBL* to be -0.40. Adjusting for measurement error in the serum level (dividing the estimate by the attenuation factor 0.37/0.45) we get a slope of -0.49 for *BL*. We rounded this to -0.50.

Scenario 1: We do not know how the individual components *DI* and *BL* relate simultaneously to stomach cancer. We first constructed a scenario in which all the effect on disease is mediated through the dietary intake. We chose a coefficient for *DI* which would lead to the observation that $\alpha_{2}$ ~ -0.50 if *DI* were omitted from the model. Approximately, $\alpha_{1}$ = $\alpha_{2}$ x var(*BL*) / ($\beta_{1}$x var(*DI*)) = −0.50 x 0.37/(0.45 x 1.14) = −0.36.

Thus our first scenario had $\alpha_{1}$=−0.36 and $\alpha_{2}$=0. We chose $\alpha_{0}$ so that that approximately 50% of the participants were controls and 50% cases. In this scenario $\alpha_{0}$=−1.01.

Scenario 2: We constructed a second scenario in which all the effect on disease is mediated through the biological marker level. Here we could apply the data from Jenab et al^39^ directly and we took $\alpha_{0}$=2.65, $\alpha_{1}$=0 and $\alpha_{2}$=−0.50.

Scenario 3:

We constructed a third scenario where the effect of disease is mediated approximately equally through the dietary intake and biological marker. We took $\alpha_{0}$=0.82, $\alpha_{1}$=−0.18 and $\alpha_{2}$=−0.25, the two coefficients being half their values in Scenarios 1 and 2.

**B5. Results**

**B5.1 Correlations between the exposure variables**

The chosen model parameters shown in Table A1 gave rise to correlations between the exposure variables, which are shown in Table A2. True dietary intake (*DI*) was correlated approximately equally with FFQ reported intake (0.70), 6x24HR reported intake (0.74), and measured serum β-cryptoxanthin level (*MBL*) (0.72). True serum lutein level (*BL*) was most strongly correlated with observed serum lutein level (0.91), and less strongly with reported dietary intake (0.58 for 6x24HR and 0.55 for FFQ).

It is noticeable that the FFQ correlation with true dietary intake was higher for β-cryptoxanthin (0.70) than for lutein (0.51), whereas for 6x24HR the correlations were similar (0.74 and 0.68 respectively). The foods with the highest levels of β-cryptoxanthin tend to be eaten in a western diet on a sporadic basis (peppers, pumpkin, winter squash, persimmons, tangerines and papayas^42^), whereas the major sources of lutein are green vegetables such as spinach and broccoli and eggs that are more regularly consumed. Relative to a 24HR, FFQs may be better able to capture intake of sporadically consumed foods but less able to capture intake of regularly consumed foods, leading to the pattern of correlations mentioned.

**B5.2 Simulation results**

Results for the three scenarios where a FFQ was the dietary instrument are shown in Table A3. The principal components method was the most powerful for the no-mediation and partial-mediation scenarios, whereas the univariate analysis of serum level was the most powerful method for the full-mediation scenario. The principal components and Howe’s method were only slightly less efficient than the univariate serum level analysis in this full-mediation scenario.

Projected sample size savings over use of the univariate dietary intake analysis were marked in the full-mediation and partial-mediation scenarios, but less so under no mediation. Thus, in the no-mediation scenario the principal components method achieved a reduction to 71% of the sample size required for the dietary intake analysis. However, in the full-mediation scenario the univariate serum analysis, principal components or Howe’s method required only about 40% of the sample size needed for a dietary intake–only analysis, and in the partial-mediation scenario about 60%.

Results where 6 24HR’s were the dietary instrument are shown in Table A4. They were similar to those obtained for the FFQ simulations. This is not surprising seeing that the correlations were similar for the two instruments (Table A2).

**B5.3 Discussion of Results**

In the β-cryptoxanthin-stomach cancer with FFQ example, the increase in power from inclusion of biomarker data was less marked than in the Lutein-ARMD with FFQ example, but still potentially useful. The smaller increase in power was partly due to the unusually high correlations between reported and true intake of β-cryptoxanthin, as inferred from the OPEN data.

Further References (in addition to those found in the main text):

39. Schatzkin A, Kipnis V, Carroll RJ, et al. A comparison of a food frequency questionnaire with a 24-hour recall for use in an epidemiological cohort study: results from the biomarker-based OPEN study. *Int J Epidemiol* 2003; **32:** 1054-1062.

40. Jenab M, Riboli E, Ferrari P, et al. Plasma and dietary carotenoid, retinol and tocopherol levels and the risk of gastric adenocarcinomas in the European Prospective Investigation into Cancer and Nutrition. *Br J Cancer* 2006; **95:** 406-415.

41. Al-Delaimy WK, Ferrari P, Slimani N, et al. Plasma carotenoids as biomarkers of intake of fruits and vegetables: individual-level correlations in the European Prospective Investigation into Cancer and Nutrition (EPIC). *Eur J Clin Nutr* 2005; **59:** 1387-1396.

42. Reports by Single Nutrients. USDA National Nutrient Database for Standard Reference, Release 20. (<http://www.ars.usda.gov/Main/docs.htm?docid=15869>) (Accessed 9/26/2007.)

Table A1: Parameters for the β-cryptoxanthin – Stomach Cancer Model

| Model | Parameter | Value* | | |
| --- | --- | --- | --- | --- |
| Biomarker-Diet^a^ | Intercept$\beta_{0}$ | 6.58 | | |
|  | Slope $\beta_{1}$ | 0.45 | | |
|  | Residual variance($\varepsilon_{BL}$) | 0.14 | | |
| Biomarker Measurement^b^ | Mean(*TBL*) | 5.30 | | |
|  | Variance(*TBL*) | 0.37 | | |
|  | Residual variance($\varepsilon_{MBL}$) | 0.08 | | |
| Dietary Intake Measurement^c^ |  | FFQ 6x24HR | | |
|  | Intercept $\gamma_{0}$ | -0.59 -0.11 | | |
|  | Slope $\gamma_{1}$ | 0.54 0.96 | | |
|  | Mean(*TDI*) | -2.81 | | |
|  | Variance(*TDI*) | 1.14 | | |
|  | Residual variance($\varepsilon_{RDI}$) | 0.34 0.86 | | |
| Disease-Diet^d^ |  | Model a | Model b | Model c |
|  | Intercept $\alpha_{0}$ | -1.01 | 2.65 | 0.82 |
|  | Coefficient $\alpha_{1}$ | -0.36 | 0.00 | -0.18 |
|  | Coefficient $\alpha_{2}$ | 0.00 | -0.50 | -0.25 |

* Biomarker level is log transformed nmol/L; Dietary intake is log transformed mg/d Parameter values are derived from data in references: Van het Hoff et al^26^, Brevik et al^28^, Al-Delaimy et al^41^, Jenab et al^40^, Dixon et al^30^, Mares et al^31^, Schatzkin et al^39^ and on unpublished data from the OPEN study^34^, as described in Appendix, Part B.

^a^ Parameters for regression of *TBL* on *TDI*

^b^ Parameters for regression of *MBL* on *TBL*

^c^ Parameters for regression of *RDI* on *TDI*

^d^ Parameters for regression of disease *D* on *TBL* and *TDI*

**Table A2: β-cryptoxanthin: Correlations Between Measurements Derived From the Chosen Model**

|  | True diet β-crypto  (*TDI*) | Reported diet β-crypto (*RDI*)  FFQ 6x24HR | True serum  β-crypto  (*TBL*) | Measured serum β-crypto (*MBL*) |
| --- | --- | --- | --- | --- |
| *TDI* | 1.00 |  |  |  |
| *RDI* FFQ  6x24HR | 0.70  0.74 | 1.00 |  |  |
| *TBL* | 0.79 | 0.55 0.58 | 1.00 |  |
| *MBL* | 0.72 | 0.50 0.53 | 0.91 | 1.00 |

Table A3: β-cryptoxanthin and Stomach Cancer (SC), With Dietary Intake Assessed by FFQ: Standardized Relative Risks (RR*), Statistical Power and Relative Sample Size** (rss) Required for Various Analysis Strategies Under Different Disease Risk Models

| Analysis Strategy |  | Disease Risk Model | | |
| --- | --- | --- | --- | --- |
|  |  | (a)  Not mediated through marker | (b)  Mediated entirely through marker | (c)  Partially mediated through marker |
| *RDI* ^a^  (univariate) | RR  Power (rss) | 0.51  0.747 (1.00) | 0.65  0.390 (1.00) | 0.58  0.540 (1.00) |
| *MBL*^b^  (univariate) | RR  Power (rss) | 0.50  0.768 (0.95) | **0.49**  **0.771 (0.39)** | 0.50  0.746 (0.62) |
| Bivariate^c^ | RR *RDI*  RR *MBL*  Power (rss) | 0.65  0.62  0.803 (0.87) | 0.90  0.52  0.690 (0.47) | 0.77  0.57  0.711 (0.67) |
| Howe^d^ (ranks) | RR  Power (rss) | 0.44  0.867 (0.73) | 0.50  0.717 (0.44) | 0.47  0.759 (0.60) |
| Principal Components^e^ | RR  Power (rss) | **0.45**  **0.877 (0.71)** | 0.52  0.728 (0.43) | **0.49**  **0.772 (0.58)** |

* Standardized relative risk is defined as the RR between the 90^th^ and 10^th^ percentiles of the distribution.

**Bold type** indicates the most powerful method among the available choices investigated.

** Sample size requirement relative to the univariate RDI analysis.

^a^ Regression of SC on *RDI*

^b^ Regression of SC on *MBL*

^c^ Regression of SC on *RDI and MBL*

^d^ Regression of SC on combination of *RDI* and *MBL* using Howe’s method

^e^ Regression of SC on combination of *RDI* and *MBL* using Principal Components

Table A4: β-cryptoxanthin and Stomach Cancer (SC), With Dietary Intake Assessed by 6 24HR’s: Standardized Relative Risks (RR*), Statistical Power and Relative Sample Size** (rss) Required for Various Analysis Strategies Under Different Disease Risk Models

| Analysis Strategy |  | Disease Risk Model | | |
| --- | --- | --- | --- | --- |
|  |  | (a)  Not mediated through marker | (b)  Mediated entirely through marker | (c)  Partially mediated through marker |
| *RDI* ^a^  (univariate) | RR  Power (rss) | 0.49  0.799 (1.00) | 0.63  0.426 (1.00) | 0.56  0.612 (1.00) |
| *MBL*^b^  (univariate) | RR  Power (rss) | 0.50  0.773 (1.07) | **0.50**  **0.769 (0.43)** | 0.50  0.756 (0.72) |
| Bivariate^c^ | RR *RDI*  RR *MBL*  Power (rss) | 0.62  0.64  0.820 (0.95) | 0.88  0.53  0.696 (0.51) | 0.74  0.59  0.732 (0.76) |
| Howe^d^ (ranks) | RR  Power (rss) | 0.43  0.866 (0.83) | 0.50  0.707 (0.50) | 0.46  0.786 (0.66) |
| Principal Components^e^ | RR  Power (rss) | **0.45**  **0.884 (0.79)** | 0.51  0.720 (0.49) | **0.48**  **0.800 (0.64)** |

* Standardized relative risk is defined as the RR between the 90^th^ and 10^th^ percentiles of the distribution.

**Bold type** indicates the most powerful method among the available choices investigated.

** Sample size requirement relative to the univariate RDI analysis.

^a^ Regression of SC on *RDI*

^b^ Regression of SC on *MBL*

^c^ Regression of SC on *RDI and MBL*

^d^ Regression of SC on combination of *RDI* and *MBL* using Howe’s method

^e^ Regression of SC on combination of *RDI* and *MBL* using Principal Components
